# Diabetes and Younger Age Are Vital and Independent Risk Factors for Acute Pancreatitis in Patients with Severe Hypertriglyceridemia

**DOI:** 10.1155/2019/2620750

**Published:** 2019-10-13

**Authors:** Qiang Li, Chaoqun Hou, Yunpeng Peng, Xiaole Zhu, Chenyuan Shi, Kai Zhang, Min Tu, Feng Guo, Dongya Huang, Yi Miao

**Affiliations:** ^1^Pancreatic Center & Department of General Surgery, The First Affiliated Hospital of Nanjing Medical University, Nanjing 210029, Jiangsu, China; ^2^Pancreas Institute of Nanjing Medical University, Nanjing 210029, Jiangsu, China

## Abstract

*Background*. The incidence of hypertriglyceridemia-induced acute pancreatitis (HIAP) is increasing worldwide, and now it is the third leading cause of acute pancreatitis in the United States. But, there are only 5% of patients with severe hypertriglyceridemia (>1000 mg/dl) which might generate acute pancreatitis. In order to explore which part of the patients is easy to develop into pancreatitis, a case-control study was performed by us to consider which patient population tend to develop acute pancreatitis in patients with severe hypertriglyceridemia. To perform a retrospective case-control study, we identified severe hypertriglyceridemia patients without AP (HNAP) and with HIAP with a fasting triglyceride level of >1000 mg/dl from The First Affiliated Hospital of Nanjing Medical University during January 1, 2014, to December 31, 2016. Baseline patient characteristics, comorbidities, and risk factors were recorded and evaluated by the univariate and multivariate logistic regression analysis for HIAP and HNAP patients. A total of 124 patients with severe hypertriglyceridemia were included in this study; of which, 62 patients were in the HIAP group and 62 were in the HNAP group. Univariate logistic regression analysis showed that there was no gender difference in both groups; however, there were more younger patients in the HIAP group than in the HNAP group (P value < 0.001), and the HIAP group had low level of high-density lipoprotein compared to the HNAP group (P<0.05). Meanwhile, the presence of pancreatitis was associated with higher level of glycemia and a history of diabetes (P<0.05). Multivariate logistic regression analysis indicated that a history of diabetes and younger age were independent risk factors for acute pancreatitis in patients with severe hypertriglyceridemia. Uncontrolled diabetes and younger age are potential risk factors in patients with severe hypertriglyceridemia to develop acute pancreatitis.

## 1. Introduction

Acute pancreatitis (AP) is a potentially life-threatening acute inflammatory disease of the pancreas. It is characterized by a systemic inflammatory response, with a growing number of hospitalizations each year, and it is associated with a mortality ranging from 3 to 30% in the world [1, 2]. In addition to gallstones and alcohol [3, 4], hypertriglyceridemia (HTG) is considered the third most common etiology of AP and accounts for 1–12% of AP cases [5–7].

It is reported that hypertriglyceridemia affects approximately one-quarter of the United States (US) population [8]. It is reported that 12% to 38% of AP patients have a history of a lipid disturbance [9]. According to the Guidelines of the American College of Gastroenterology and the Endocrine Society, high TG levels (≥1000 mg/dL) should be considered as a risk factor for developing acute pancreatitis [10, 11]. Elevated serum triglycerides (TGs) have been positively associated with the risk of AP, but the exact mechanism remains nebulous. As diabetes represents metabolic disorders, the endocrine function of the pancreas is affected, altering the carbohydrate and lipid metabolism. Lai et al. [12] reported that Chinese patients with diabetes are at elevated risk of AP in a population-based cohort study. Another study proposed that the risk of AP in diabetic patients can be reduced with better blood sugar control [13]. Furthermore, another recent study reported that increased lipid peroxidation associated with chronic hyperglycemia may be a key event in the pathogenesis of AP [14]. The present study showed that not all patients with HTG would develop AP, and the risk factors associated with AP in severe HTG patients are not well clarified. More importantly, a few studies have evaluated the development of AP in patients with severe HTG. Given the abovementioned gaps and information, we conduct a retrospective case-control study to assess the risk factors of HTG-AP.

## 2. Patients and Methods

The First Affiliated Hospital of Nanjing Medical University is an integrated healthcare delivery system in Eastern China. Data available for this study included demographic data, diagnoses, medication dispensing, and laboratory results. The hospital has an electronic health medical record system and a paper-based medical records library, which allow access to more detailed information and was included in this study.

**Figure 1:**
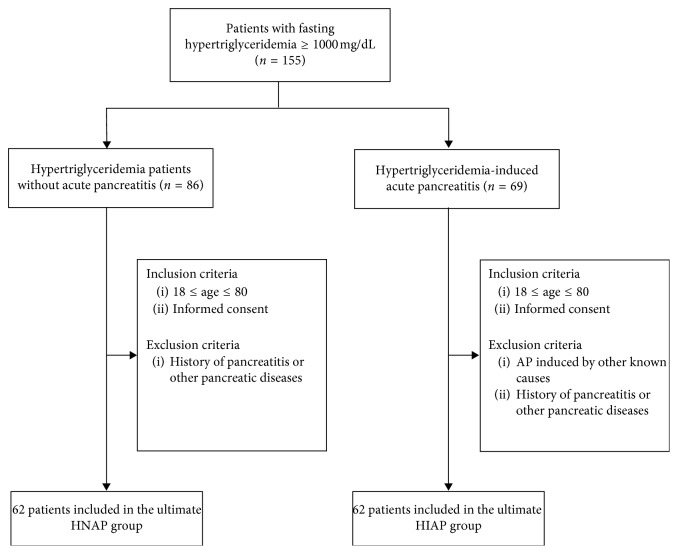
Diagram of the study flow.

HNAP and HIAP patients with a fasting triglyceride level of >1000 mg/dl were retrospectively analyzed in The First Affiliated Hospital of Nanjing Medical University from January 1, 2014, to December 31, 2016. Among these patients, those with biliary pancreatitis, alcoholic pancreatitis, idiopathic causes, traumatic pancreatitis, and other undefined recorded causes were excluded from our study. The diagnosis of HIAP was made if the patients had AP with the serum total triglyceride (TG) level of >11.3 mmol/L (1000 mg/dl). The AP diagnosis was performed based on the following features: typical upper abdominal pain (persistent, severe, radiating pain), laboratory tests (amylase and lipase), and evidence of pancreatitis upon abdominal imaging [14]. CT scans were performed on all patients upon admission to the hospital to differentiate HIAP from other medical conditions. During the study period, a total of 155 patients were diagnosed with hypertriglyceridemia. According to our criteria, 124 patients with severe hypertriglyceridemia were included in this study; of which, 62 patients were in the HIAP group and 62 patients were in the HNAP group (Figure 1).

The information on demographic data, history of diabetes, blood pressure, history of alcohol and cigarettes, and biochemical data was carefully collected. Data regarding deaths, length of hospital stay, and intensive care unit (ICU) admission were recorded and analyzed.

All the patients whose data were collected for this study's purpose provided written informed consent for the acquisition, analysis, and publishing of the anonymized data collected during their hospital admission.

### 2.1. Statistical Analysis

*P* < 0.05 was considered statistically significant, and all statistical analyses were performed by using SPSS17.0 software package. Continuous variables are expressed as mean ± SE, and comparisons between groups were performed by using the *t*-test. Categorical variables were compared between groups by using the Pearson *χ*^2^ test.

## 3. Results

### 3.1. Baseline Characteristics of the Study Sample

The baseline characteristics of hypertriglyceridemia patients with or without AP are summarized in Table 1; a total of 124 patients with severe hypertriglyceridemia were included in this study. Differences between groups were analyzed by the chi-squared test or Fisher exact tests. Age, gender, weight, blood pressure, and mortality and survival rate were well balanced between the groups. The HIAP group were more likely to have a significantly higher proportion of diabetes (*P*=0.001) and higher fasting glycemia (*P* < 0.001) than the HNAP group. Although there is no statistical difference, a high percentage of smoking history and drinking history was also observed in the HIAP group.

### 3.2. Univariate Logistic Regression

To investigate the risk factors contributing HIAP occurrence, we examined the potential variables and analyzed them by univariate analysis, as shown in Table 2. Univariate analysis identified that younger age (OR 0.901, 95% CI 0.931–4.22; *P* < 0.001) and lower HDL-c (OR 0.995, 95% CI 0.992–0.998; *P*=0.003) were significantly associated with HIAP occurrence. In addition, the presence of pancreatitis was associated with higher hepatic biochemical indexes, such as GGT, LDH, AST, ALT, and alkaline phosphatase. Furthermore, a history of diabetes was more common in patients with HIAP (OR 4.321, 95% CI 1.683–11.096; *P*=0.002), and higher fasting glycemia (OR 1.126, 95% CI 1.040–1.218; *P*=0.003) was associated with the presence of pancreatitis.

### 3.3. Multivariate Logistic Regression

A binomial logistic regression was performed to ascertain the effects of independent variables on the likelihood that patients had acute pancreatitis with severe hypertriglyceridemia.

Introducing the variables with *P* value <0.05 and higher clinical relevance in univariate analysis into multivariate analysis (Table 3), the result indicated that diabetes (OR 6.41, 95% CI 1.4933–27.496; *P*=0.012) and lower age (OR 0.95, 95% CI 0.9–0.9992; *P*=0.047) were associated with an increased likelihood of developing HIAP.

### 3.4. ICU Admission and Length of Hospital Stay

Comparison between HIAP patients with diabetes (*n* = 22) and those without (*n* = 40) was performed. ICU admission was more frequent in the HIAP patients with diabetes than those without, and obviously, the difference was statistically significant (*χ*^2^ = 5.573, *P*=0.018) (Table 4). Similarly, median length of hospital stay in HIAP patients with diabetes was 15.2 days (9.0–18.3) vs. 9.5 days (5.3–16.0) in the nondiabetes group (*P*=0.06) (Table 5).

## 4. Discussion

In the present study, univariate and multivariate analyses identified that a history of diabetes and younger age were independent risk factors for acute pancreatitis in patients with severe hypertriglyceridemia. Moreover, we found that diabetes can influence the management of patients with HIAP, increase ICU admission rate, and prolong hospital length of stay. To our knowledge, this is the first case-control study to examine the risk factors in people with severe hypertriglyceridemia in China.

The mechanism by which HTG leads to acute pancreatitis has not been clarified so far; however, several pathogenetic theories have been proposed. Bhatia et al. [15, 16] proposed that HTG can impair circulatory blood flow in the pancreatic, leading to an increase in affinity between hemoglobin and oxygen, which may induce tissue hypoxia. Poonuru et al. [17] demonstrated that triglycerides in the pancreas are broken down by pancreatic lipases into free fatty acids and excessively produced free fatty acids accumulate in the pancreatic microcirculation, leading to obstruction and ischemia, which can induce acute pancreatitis. This result can also increase the acidity of the pancreas, which makes the pancreas prone to acute inflammation through activation of trypsinogen. In our study, we found that a history of smoking and drinking was more common in HIAP patients. A recent comprehensive study analyzing 2810 patients demonstrated that cigarette smoking and drinking were associated with non-gallstone-related pancreatitis, including hypertriglyceridemia-induced acute pancreatitis [18]. Another study noted that cigarette smoking might contribute to hyperlipidemic acute pancreatitis recurrence [19]. Future studies are needed to clarify possible changes in the metabolic and molecular characteristics of HIAP related to tobacco use and drinking.

HTG has also been shown to exacerbate other experimental models of acute pancreatitis [20]. Diabetes can lead to lipid metabolism disorder, which aggravates the process and induces pancreatitis, but further experiments are needed to support this standpoint. A recent study of 784 patients with acute pancreatitis also reported that 67% patients with diabetes or impaired glucose tolerance had a secondary risk factor for hypertriglyceridemia-induced acute pancreatitis [21]. The most interesting finding of our study is that diabetes and younger age were independently associated with developing HIAP. Furthermore, the prevalence of ICU admission and median hospital days were higher in HIAP patients with diabetes. These results are consistent with those of previous studies.

There are some special features of HTG-AP different from AP of other etiologies that are frequently discussed. Some older studies proposed that HTG may play a critical role in the development of respiratory failure and other severe complications associated with acute pancreatitis [22, 23]. A French cohort study showed that the severity of HTG-induced AP was higher than that of AP induced by gallstones or alcohol [24]. Furthermore, HTG-induced AP is found to have a more severe prognosis, longer hospitalization, and more frequent pancreatic necrosis in patients [25]. In addition, an important practical point from one study showed that some AP patients manifested “normal” serum amylase activity levels although they had a raised urine amylase activity. Thus, the normal serum amylase activity does not exclude the diagnosis of acute pancreatitis in the presence of severe hypertriglyceridemia [22, 26, 27].

To date, the exact pathophysiological mechanism of HTG-induced AP still remains unclear and is a topic of controversial discussion. However, further research efforts in this area will be needed to spread more light into the risk factor of hypertriglyceridemia-induced acute pancreatitis.

This study has several limitations. First, our study used a retrospective design, which did not make dynamic analysis of patients' data. Second, there is no information regarding additional factors that may have contributed to pancreatic-related events such as GLP-1 receptor agonists and DPP-4 inhibitors [28, 29]. The duration of diabetes and HTG was not considered in this study. Third, biochemistry data such as hemoglobin A1C, C-peptide, and insulin levels were not collected and analyzed in all patients. These data are important because they affect the severity of acute pancreatitis. Finally, sample size was small in our study; hence, the conclusion needs to be confirmed further in a large-scale prospective clinical observation.

## 5. Conclusions

In conclusion, we demonstrated that diabetes and younger are vital and independent risk factors for acute pancreatitis in patients with severe hypertriglyceridemia. Moreover, the status of diabetes can aggravate the development of HIAP. These findings suggest that severe hypertriglyceridemia in young diabetic patients require more intensive pharmacologic therapeutic strategies, and HIAP patients who have diabetes should have more aggressive management. Further major clinical studies and animal experiments are needed to establish the influence of diabetes towards severity and mortality in HIAP.

## Figures and Tables

**Table 1:** Baseline characteristics of the patients.

	HNAP (*n* = 62)	HIAP (*n* = 62)	*P* value
Age (years)			0.25
≥45	23 (37%)	17 (27%)	
<45	39 (63%)	45 (73%)	
Gender			0.09
Male	46 (74%)	37 (60%)	
Female	16 (26%)	25 (40%)	
History of drinking			0.09
Yes	11 (18%)	19 (31%)	
No	51 (82%)	43 (69%)	
History of smoking			0.08
Yes	10 (16%)	18 (29%)	
No	52 (84%)	44 (71%)	
Weight (kg)			0.58
≥70	35 (56%)	38 (61%)	
<70	27 (44%)	24 (39%)	
Hypertension			0.83
Yes	16 (26%)	15 (24%)	
No	46 (74%)	47 (76%)	
History of diabetes			0.001
Yes	7 (11%)	22 (35%)	
No	55 (89%)	40 (65%)	
Fasting glycemia (mmol/l)			<0.001
≥6.1	24 (39%)	44 (71%)	
<6.1	38 (61%)	18 (29%)	

HNAP, hypertriglyceridemia patients without acute pancreatitis; HIAP, hypertriglyceridemia-induced acute pancreatitis.

**Table 2:** Univariate logistic regression analysis.

	*N*	OR	*z*	*P* value	LOR	UOR
Gender	124	1.672	1.33	0.183	0.784	3.563
Age	124	0.931	−4.22	<0.001	0.901	0.963
History of drinking	124	2.049	1.66	0.097	0.879	4.775
History of smoking	124	1.752	1.28	0.2	0.743	4.130
Weight	124	1.016	1.16	0.246	0.989	1.043
Hypertension	124	1.090	0.21	0.836	0.483	2.458
Length of hospital stays	124	1.051	1.67	0.095	0.991	1.115
History of diabetes	124	4.321	3.04	0.002	1.683	11.096
Fasting glycemia	124	1.126	2.94	0.003	1.040	1.218
Lipoprotein a	111	1.002	1.03	0.302	0.998	1.007
LDL-c	112	1.001	0.5	0.619	0.998	1.004
HDL-c	112	0.995	−2.97	0.003	0.992	0.998
Total cholesterolemia	117	0.999	−0.53	0.598	0.996	1.003
Direct bilirubin	124	1.011	1.47	0.142	0.996	1.027
Triglycerides	124	0.998	−0.43	0.668	0.988	1.008
Alkaline phosphatase	124	1.044	5.76	<0.001	1.029	1.060
GGT	123	1.113	4.44	<0.001	1.062	1.167
LDH	124	1.011	5.59	<0.001	1.007	1.015
AST	122	1.125	5.26	<0.001	1.076	1.176
ALT	122	0.993	−2.04	0.041	0.987	1.000

LDL-c, low-density lipoprotein cholesterol; HDL-c, high-density lipoprotein cholesterol; GGT, gamma-galactosyltransferase; LDH, lactate dehydrogenase; AST, aspartate transaminase; ALT, alanine transaminase; OR, odds ratio.

**Table 3:** Multivariate logistic regression analysis.

	OR	*z*	*P* value	LOR	UOR
Age	0.95	−1.99	0.047	0.9	0.9992
History of drinking	1.88	0.77	0.441	0.3763	9.407
History of diabetes mellitus	6.41	2.5	0.012	1.4933	27.496
Fasting glycemia	1.09	1.85	0.064	0.9952	1.1883
HDL-c	1.01	1.05	0.292	0.9977	1.0077

HDL-c, high-density lipoprotein cholesterol; OR, odds ratio.

**Table 4:** ICU admission.

Monocentric	HIAP		*χ* ^2^	*P* value
	Nondiabetes	With diabetes		
ICU	7	10	5.573	0.018
Non-ICU	33	12		

HIAP, hypertriglyceridemia-induced acute pancreatitis; ICU, intensive care unit.

**Table 5:** Length of hospital stay.

		Sample	Length of hospital stay	*z*	*P* value
HIAP	Nondiabetes	40	9.5 (5.3, 16.0)	0.614	0.006
	Diabetes	22	15.2 (9.0, 18.3)		

HIAP, hypertriglyceridemia-induced acute pancreatitis.

## Data Availability

All data in our study are available from the corresponding author upon reasonable request.

## References

[1] Johnson C. D., Besselink M. G., Carter R. (2014). Acute pancreatitis. *BMJ*.

[2] Wu B. U., Banks P. A. (2013). Clinical management of patients with acute pancreatitis. *Gastroenterology*.

[3] Working Party of the British Society of Gastroenterology (2005). UK guidelines for the management of acute pancreatitis. *Gut*.

[4] Forsmark C. E., Baillie J. (2007). AGA Institute technical review on acute pancreatitis. *Gastroenterology*.

[5] Athyros V. G., Giouleme O. I., Nikolaidis N. L. (2002). Long-term follow-up of patients with acute hypertriglyceridemia-induced pancreatitis. *Journal of Clinical Gastroenterology*.

[6] Fortson M. R., Freedman S. N., Webster P. D (1995). Clinical assessment of hyperlipidemic pancreatitis. *American Journal of Gastroenterology*.

[7] Zheng Y., Zhou Z., Li H (2015). A multicenter study on etiology of acute pancreatitis in Beijing during 5 years. *Pancreas*.

[8] James B., Deborah A., Daniel H., Julia C. (2016). The clinical relevance of omega-3 fatty acids in the management of hypertriglyceridemia. *Lipids in Health & Disease*.

[9] Toskes P. P. (1990). Hyperlipidemic pancreatitis. *Gastroenterology Clinics of North America*.

[10] Berglund L., Brunzell J. D., Goldberg A. C. (2012). Evaluation and treatment of hypertriglyceridemia: an Endocrine Society clinical practice guideline. *The Journal of Clinical Endocrinology & Metabolism*.

[11] Tenner S., Baillie J., Dewitt J., Vege S. S. (2013). American College of Gastroenterology guideline: management of acute pancreatitis. *American Journal of Gastroenterology*.

[12] Lai S.-W., Muo C.-H., Liao K.-F., Sung F.-C., Chen P.-C. (2011). Risk of acute pancreatitis in type 2 diabetes and risk reduction on anti-diabetic drugs: a population-based cohort study in Taiwan. *American Journal of Gastroenterology*.

[13] Antonio G. P., Schlienger R. G., García R. L. A. (2010). Acute pancreatitis in association with type 2 diabetes and antidiabetic drugs. *Diabetes Care*.

[14] Steer M. L. (1999). Early events in acute pancreatitis. *Best Practice & Research Clinical Gastroenterology*.

[15] Bhatia M. (2005). Inflammatory response on the pancreatic acinar cell injury. *Scandinavian Journal of Surgery*.

[16] Ditzel J., Hau C., Daugaard N. (1977). Effect of the diphosphonate EHDP on plasma inorganic phosphate and hemoglobin oxygen affinity of diabetic and healthy subjects. *Advances in Experimental Medicine and Biology*.

[17] Poonuru S., Pathak S. R., Vats H. S., Pathak R. D. (2011). Rapid reduction of severely elevated serum triglycerides with insulin infusion, gemfibrozil and niacin. *Clinical Medicine & Research*.

[18] Setiawan V. W., Pandol S. J., Porcel J. (2016). Prospective study of alcohol drinking, smoking, and pancreatitis. *Pancreas*.

[19] Xiang J.-X., Hu L.-S., Liu P. (2017). Impact of cigarette smoking on recurrence of hyperlipidemic acute pancreatitis. *World Journal of Gastroenterology*.

[20] Czakó L., Szabolcs A., Vajda Á. (2007). Hyperlipidemia induced by a cholesterol-rich diet aggravates necrotizing pancreatitis in rats. *European Journal of Pharmacology*.

[21] Charlesworth A., Steger A., Crook M. A. (2015). Acute pancreatitis associated with severe hypertriglyceridaemia; a retrospective cohort study. *International Journal of Surgery*.

[22] Warshaw A. L., Lesser P. B., Rie M., Cullen D. J. (1975). The pathogenesis of pulmonary edema in acute pancreatitis. *Annals of Surgery*.

[23] Kimura T., Toung J. K., Margolis S., Permutt S., Cameron J. L. (1979). Respiratory failure in acute pancreatitis: a possible role for triglycerides. *Annals of Surgery*.

[24] Linares C. L., Pelletier A. L., Czernichow S. (2008). Acute pancreatitis in a cohort of 129 patients referred for severe hypertriglyceridemia. *Pancreas*.

[25] Baranyai T., Terzin V., Vajda Á., Wittmann T., Czakó L. (2012). Hypertriglyceridemia causes more severe course of acute pancreatitis. *Clinical Lipidology*.

[26] Orebaugh S. L. (1994). Normal amylase levels in the presentation of acute pancreatitis. *The American Journal of Emergency Medicine*.

[27] Shah A. M., Eddi R., Kothari S. T., Maksoud C., DiGiacomo W. S., Baddoura W. (2010). Acute pancreatitis with normal serum lipase: a case series. *Journal of the Pancreas*.

[28] Faillie J. L., Babai S., Crépin S (2014). Pancreatitis associated with the use of GLP-1 analogs and DPP-4 inhibitors: a case/non-case study from the French Pharmacovigilance Database. *Acta Diabetologica*.

[29] Lando H., Alattar M., Dua A. (2012). Elevated amylase and lipase levels in patients using glucagonlike peptide-1 receptor agonists or dipeptidyl-peptidase-4 inhibitors in the outpatient setting. *Endocrine Practice*.

